# Non-alcoholic/Metabolic-Associated Fatty Liver Disease and *Helicobacter pylori* Additively Increase the Risk of Arterial Stiffness

**DOI:** 10.3389/fmed.2022.844954

**Published:** 2022-02-25

**Authors:** Ji Min Choi, Hyo Eun Park, Yoo Min Han, Jooyoung Lee, Heesun Lee, Su Jin Chung, Seon Hee Lim, Jeong Yoon Yim, Goh Eun Chung

**Affiliations:** ^1^Division of Gastroenterology and Hepatology, Department of Internal Medicine, Seoul National University Hospital Healthcare System Gangnam Center, Seoul, South Korea; ^2^Division of Cardiology, Department of Internal Medicine, Seoul National University Hospital Healthcare System Gangnam Center, Seoul, South Korea

**Keywords:** *Helicobacter*, hepatic steatosis, arterial stiffness, risk, atherosclerosis

## Abstract

**Background:**

Non-alcoholic fatty liver disease (NAFLD) and *Helicobacter pylori* (*Hp*) infection have a close association with an increased risk of cardiovascular disease. Metabolic dysfunction-associated fatty liver disease (MAFLD) is characterized by metabolic dysfunction in NAFLD. We investigated the synergistic effects of NAFLD/MAFLD and *Hp* infection on the risk of arterial stiffness in an asymptomatic population.

**Methods:**

We included individuals who underwent abdominal ultrasonography, anti-*Hp* IgG antibody evaluations and cardio-ankle vascular index (CAVI) during health screening tests between January 2013 and December 2017. Arterial stiffness was defined using CAVI. A logistic regression model was used to analyze the independent and synergistic effects of NAFLD/MAFLD and *Hp* infection on the risk of arterial stiffness.

**Results:**

Among 3,195 subjects (mean age 54.7 years, 68.5% male), the prevalence of increased arterial stiffness was 36.4%. In the multivariate analysis, subjects with NAFLD but without *Hp* infection and those with both NAFLD and *Hp* infection had a significantly higher risk of increased arterial stiffness [odds ratio (OR) 1.61, 95% confidence interval (CI) 1.15–2.26, and OR 2.23, 95% CI 1.63–3.06, respectively], than subjects without *Hp* infection and NAFLD. Regarding MAFLD, *Hp* infection additively increased the risk of arterial stiffness in subjects with MAFLD (OR 2.13, 95% CI 1.64–2.78).

**Conclusions:**

An interactive effect of *Hp* infection on the risk of arterial stiffness in individuals with NAFLD/MAFLD was observed. *Hp* infection additively increases the risk of arterial stiffness in subjects with NAFLD or MAFLD.

## Introduction

Non-alcoholic fatty liver disease (NAFLD) is a substantial public health burden, with a prevalence of up to 25% of global population (1). NAFLD is closely associated with various metabolic conditions, including obesity, dyslipidemia, type 2 diabetes, and cardiovascular disease (2). In particular, NAFLD is known to be related to arterial stiffness, a surrogate marker of systemic atherosclerosis (3). Recently, in recognition of the close association between NAFLD and metabolic dysfunction, a new term “metabolic (dysfunction)-associated fatty liver disease (MAFLD)” has been introduced, and studies on its clinical significance have been conducted (4, 5).

*Helicobacter pylori (Hp)* is a gram-negative microorganism that infects more than half of the global population (6). While *Hp* is considered to cause many gastrointestinal diseases, such as chronic gastritis, peptic ulcers and gastric cancer (7, 8), its role in extragastric diseases, including metabolic syndrome and hematological and cardiovascular diseases, has also been studied (9). The association between *Hp* infection and cardiovascular risk factors or arterial stiffness has been reported (10–12). Some mechanisms, including chronic inflammation, free radical formation, and the immune response, may be the link between chronic *Hp* infection and atherogenesis (13). Also, *Hp* infection has been associated with insulin resistance (14), which is closely linked with increased arterial stiffness (15, 16).

Since both *Hp* infection and NAFLD are involved in the pathogenesis of insulin resistance and share proinflammatory conditions (17), they are known to be independently associated with arterial stiffness. Based on this background, we hypothesized that the combination of NAFLD and *Hp* infection increases the risk of arterial stiffness. Little has been reported about the association of MAFLD with arterial stiffness. Thus, we aimed to investigate the interactive effects of NAFLD/MAFLD and *Hp* infection on arterial stiffness in an asymptomatic population.

## Methods

### Study Population

**Figure 1:**
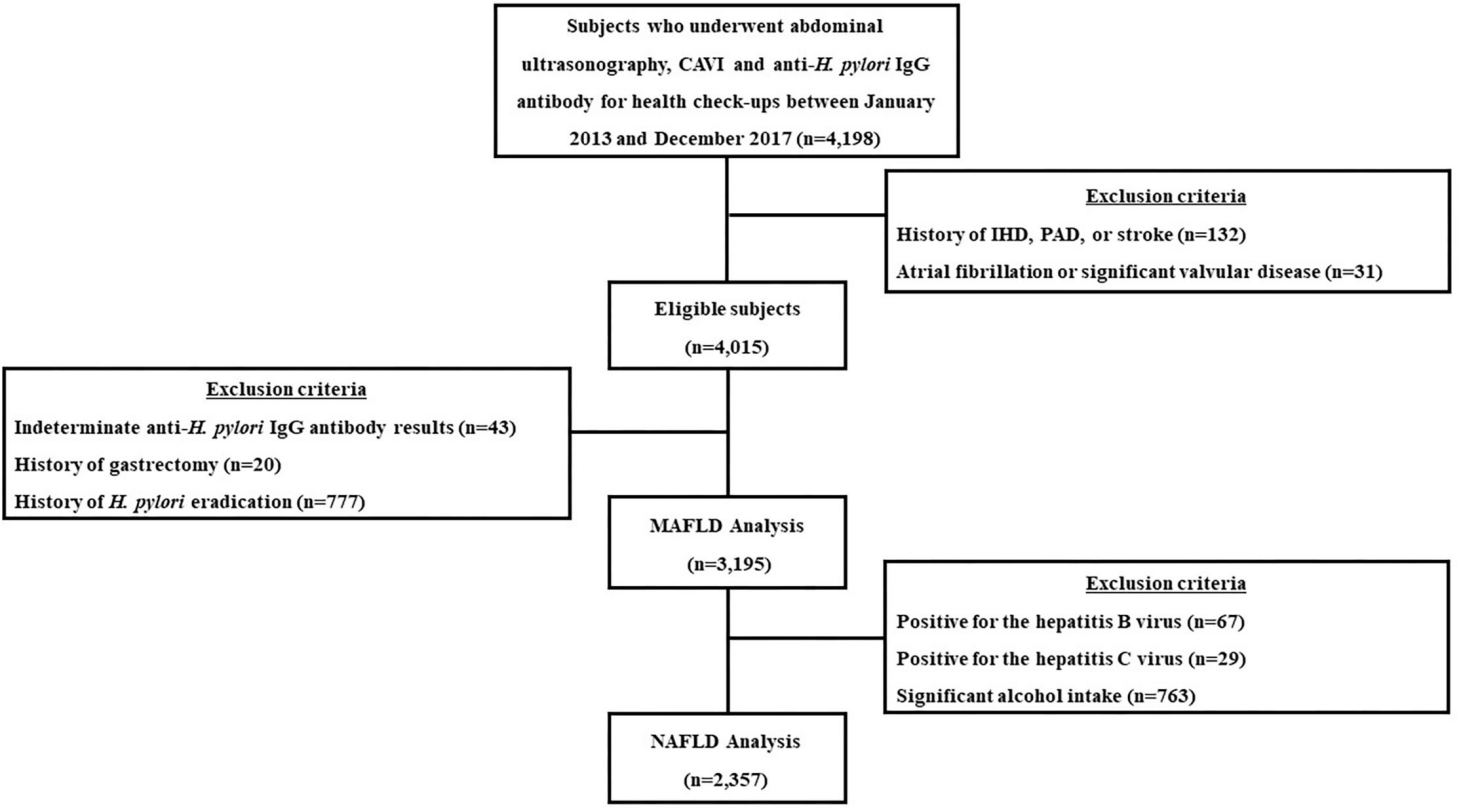
A flow diagram of the study population. CAVI, cardio-ankle vascular index; *H. pylori, Helicobacter pylori*; n, number; IHD, ischemic heart disease; PAD, peripheral artery disease; MAFLD, metabolic-dysfunction associated fatty liver disease; NAFLD, nonalcoholic fatty liver disease.

This retrospective cohort study included individuals who underwent routine health check-ups, including abdominal ultrasonography, anti-*Hp* IgG antibody testing and cardio-ankle vascular index (CAVI) evaluations, on the same day at the Seoul National University Hospital Healthcare System Gangnam Center from January 2013 to December 2017. The subjects were mostly symptom-free and willfully underwent examinations either voluntarily or were supported by their employers for the check-ups. Among the total eligible subjects, those who met the following criteria were excluded from the study: a prior history of ischemic heart disease, peripheral artery disease or stroke (*n* = 132), significant arrhythmia or valvular heart disease (*n* = 31), indeterminate anti-*Hp* IgG antibody results (*n* = 43), and a history of gastrectomy (*n* = 20) or *Hp* eradication (*n* = 777) (12). Finally, 3,195 subjects were included in the analysis. For the NAFLD analysis, subjects who displayed any potential cause of chronic liver disease were additionally excluded: 67 were positive for the hepatitis B virus, 29 were positive for the hepatitis C virus, and 763 had significant alcohol intake (>20 g/day). As a result, 2,357 subjects were included in the NAFLD analysis (Figure 1).

The study protocol followed the guidelines of the Declaration of Helsinki of 1975 and its revision in 1983. The protocol was approved by the Institutional Review Board of Seoul National University Hospital (No. 2005-051-1121). The requirement for informed consent was waived by the board, as researchers only accessed and analyzed deidentified data.

### Measurement of Anthropometric and Laboratory Parameters

The methods employed in this study have been described previously in detail (18). Anthropometric and laboratory parameters were measured on the same day as the health check-ups. Body weight and height were measured using a digital scale, and body mass index (BMI) was calculated by dividing weight (kg) by the squared value of height (m^2^). Waist circumference (WC) was measured at the midpoint between the lower costal margin and the anterior superior iliac crest by a well-trained person using a tape measure. Data regarding past medical history, comorbidities, and medication history were obtained using subject-recorded questionnaires. Based on smoking status, subjects were categorized as never or ever-smokers. The amount of alcohol each patient consumed was calculated. Blood pressure was measured at least twice, and mean values of the measurements were recorded. Hypertension was defined as blood pressure ≥140/90 mmHg or receiving antihypertensive medications. Diabetes was defined as a fasting blood glucose level ≥126 mg/dL or glycated hemoglobin A1c (HbA1c) level ≥6.5% or treatment with glucose-lowering agents. Dyslipidemia was defined as a total cholesterol level ≥240 mg/dL and/or triglyceride level ≥200 mg/dL and/or high-density lipoprotein (HDL) cholesterol level <40 mg/d or the use of anti-dyslipidemic medications (19).

All blood samples were collected after a 12-h overnight fast. Laboratory tests included serum fasting glucose, total cholesterol, triglyceride, HDL cholesterol, HbA1c, and high-sensitivity C-reactive protein (hs-CRP) levels. All of these tests were performed using standard laboratory methods. The diagnosis of *Hp* infection was based on the results of a serum anti-*Hp* IgG antibody test using a commercially available chemiluminescent microparticle immunoassay kit (Immulite® 2000 CMIA, Siemens, Germany) as described previously (12). Values >1.10 IU/mL were considered positive (20). The *Hp* IgG kit has a sensitivity of 91% and a specificity of 100% (6).

### Measurement of NAFLD/MAFLD and Advanced Fibrosis

Abdominal ultrasonography (Acuson Sequoia 512; Siemens, Mountain View, CA) was performed to diagnose fatty liver by experienced radiologists who were unaware of the clinical information of the individuals. Fatty liver was diagnosed based on characteristic ultrasonographic findings consistent with a “bright liver” and evident contrast between hepatic and renal parenchyma, focal sparing, vessel blurring, and narrowing of the lumen of the hepatic veins (21). MAFLD was diagnosed as the presence of hepatic steatosis with 1 or more of the following: (1) overweight or obese (BMI ≥ 23 kg/m^2^) (2) diabetes mellitus (3) at least 2 metabolic risk abnormalities. Metabolic risk abnormalities consisted of (1) WC ≥ 90 cm for men and 80 ≥ cm for women, (2) blood pressure ≥ 130/85 mmHg or specific drug treatment, (3) fasting plasma triglycerides ≥ 150 mg/dl or specific drug treatment, (4) plasma HDL-cholesterol <40 mg/dl for men and <50 mg/dl for women or specific drug treatment, (5) prediabetes (fasting glucose 100–125 mg/dl or hemoglobin A1c 5.7–6.4%, (6) homeostasis model assessment of insulin resistance score ≥ 2.5, (7) plasma hs-CRP level > 2 mg/L (5, 22).

For subjects with NAFLD or MAFLD, the Fibrosis-4 (FIB-4) index was used as a surrogate marker for advanced liver fibrosis. FIB-4 was calculated as age (years) × AST (U/L)/platelet (10^9^/L) × √ALT (U/L) and three risk categories (low, intermediate, high) for FIB-4 were based on the 2 cut points (1.30 and 2.67).We used the lower cutoff of FIB-4 index <1.30 to exclude advanced liver fibrosis (23).

### Assessment of Arterial Stiffness Using CAVI

CAVI was measured using a VaSera VS-1000 (Fukuda Denshi Co Ltd, Tokyo, Japan) as described in previous studies to evaluate arterial stiffness (3, 12, 24). Briefly, the brachial pulse pressure was measured using an automated cuff oscillometer in seated individuals after 5 min of rest. The average value of two measurements was calculated to determine the systolic and diastolic pressures and pulse pressure. While the individuals were resting in a supine position, the cuffs were applied to ankles and both upper arms. After a 10 min rest period, the measurement was recorded. A phonocardiogram used for the detection of heart sounds was placed over the right sternum between the second intercostal spaces, and electrocardiogram electrodes were applied on both wrists. The pulse wave velocity was calculated as the vascular length (L) divided by the time (T) required for the pulse wave to propagate from the aortic valve to the ankle. Because the initiation of blood release from the aortic valve is difficult to identify based on the opening sound of the valve, T is difficult to determine; thus, the T value was calculated by summing the interval between the initiation of the brachial pulse waveform and the initiation of the ankle pulse waveform and the interval between the closing sound of the aortic valve and the notch of the brachial pulse waveform. Measurements were performed by a well-trained staff member. The CAVI was determined using the following equation:


$$\begin{array}{l} \text{CAVI}=a\left[\left(\frac{2\rho }{\Delta P}\right)\;\times \;ln\left(\frac{Ps}{Pd}\right)\;\times PWV^{2\;}\right]+b \end{array}$$

where *P*s and *P*d are the systolic and diastolic blood pressures, respectively, Δ*P* is *Ps*–*Pd*, ρ is the blood density, and *a* and *b* are constants. The mean values of the left and right CAVI were used. We used a cutoff value of 8 to define increased arterial stiffness based on previous studies (12, 25, 26).

### Statistical Analysis

Continuous variables with a normal distribution are reported as the means ± SD or medians (with interquartile ranges) and categorical variables are reported as numbers and percentages. To test for normality, the Kolmogorov-Smirnov test and the normal Q-Q plots were used. Student's *t*-test was used when the data were normally distributed, and Mann–Whitney *U*-test was used otherwise. The differences between nominal variables were compared with the chi-square test or Fisher exact test. We divided participants into four groups according to the presence of NAFLD/MAFLD and/or *Hp* infection. A logistic regression analysis was utilized to analyze the association between NAFLD or MAFLD with *Hp* infection and increased arterial stiffness after adjusting for potential confounders. Among variables with a *P* <0.05 in the univariate analysis, those with clinical importance were subjected to multivariate analyses. All statistical analyses were performed using SPSS 22.0 (SPSS Inc., Chicago, IL, USA), and *P* <0.05 were considered statistically significant.

## Results

### Study Population

The mean age of 3,195 subjects was 54.7 years, and the proportion of males was 68.5%. The prevalence rate of increased arterial stiffness (CAVI ≥ 8) was 36.4%. Table 1 shows the baseline characteristics of the study population according to the presence of NAFLD or MAFLD. Individuals with NAFLD or MAFLD have been observed more frequently in male (70.3 vs. 51.3% in NAFLD vs. no-NAFLD and 78.8 vs. 59.3% in MAFLD vs. no-MAFLD, respectively, *P* <0.001), and ever smokers (44.0 vs. 32.2% in NAFLD vs. no-NAFLD and 53.9 vs. 38.9% in MAFLD vs. no-MAFLD, respectively, *P* <0.001). In individuals with NAFLD or MAFLD, traditional risk factors of atherosclerosis were significantly more common compared to those without NAFLD or MAFLD: hypertension (54.3 vs. 34.9% in NAFLD vs. no-NAFLD and 59.6 vs. 37.6% in MAFLD vs. no-MAFLD, respectively, *P* <0.001), diabetes (25.9 vs. 9.0% in NAFLD vs. no-NAFLD and 26.7 vs. 8.9% in MAFLD vs. no-MAFLD, respectively, *P* <0.001), and dyslipidemia (64.9 vs. 41.3% in NAFLD vs. no-NAFLD and 64.8 vs. 40.3% in MAFLD vs. no-MAFLD, respectively, *P* <0.001). In addition, most of the body measurements and laboratory results (including WC, BMI, systolic or diastolic blood pressure, triglyceride, HDL cholesterol, fasting glucose, and HbA1c levels) were less favorable in terms of metabolism in individuals with NAFLD or MAFLD (*P* <0.001). The prevalence of increased arterial stiffness was significantly higher in both patients with NAFLD and MAFLD than those without NAFLD/MAFLD (41.2 vs. 32.1% in NAFLD vs. no-NAFLD and 40.8 vs. 32.5% in MAFLD vs. no-MAFLD, respectively).

**Table 1:** Comparison of baseline characteristics according to NAFLD and MAFLD.

	**NAFLD analysis**			**MAFLD analysis**		
	**No NAFLD**	**NAFLD**	***P*-value**	**No MAFLD**	**MAFLD**	***P*-value**
	**(*N* = 1,252)**	**(*N* = 1,105)**		**(*N* = 1,699)**	**(*N* = 1,496)**	
Age (years)	54.6 ± 9.9	55.5 ± 8.9	0.032	54.3 ± 9.6	55.0 ± 8.7	0.035
Male, *n* (%)	642 (51.3)	777 (70.3)	<0.001	1008 (59.3)	1179 (78.8)	<0.001
Smoker, *n* (%)	403 (32.2)	486 (44.0)	<0.001	661 (38.9)	806 (53.9)	<0.001
Hypertension, *n* (%)	437 (34.9)	600 (54.3)	<0.001	638 (37.6)	891 (59.6)	<0.001
Diabetes, *n* (%)	113 (9.0)	286 (25.9)	<0.001	151 (8.9)	400 (26.7)	<0.001
Dyslipidemia, *n* (%)	517 (41.3)	717 (64.9)	<0.001	684 (40.3)	970 (64.8)	<0.001
BMI (kg/m^2^)	22.8 ± 2.9	25.5 ± 3.3	<0.001	23.0 ± 2.8	25.9 ± 3.1	<0.001
WC (cm)	82.3 ± 8.4	90.3 ± 8.7	<0.001	83.0 ± 8.3	91.3 ± 8.2	<0.001
SBP (mmHg)	123.8 ± 13.7	129.0 ± 13.5	<0.001	124.4 ± 13.5	130.1 ± 13.5	<0.001
DBP (mmHg)	80.3 ± 9.6	84.5 ± 9.3	<0.001	81.0 ± 9.6	86.0 ± 9.3	<0.001
Total cholesterol (mg/dL)	196.6 ± 37.3	199.0 ± 38.4	0.131	196.1 ± 36.6	198.0 ± 33.3	0.150
Triglyceride (mg/dL)^a^	71 (50,100)	118 (81,161)	<0.001	71 (50,102)	120 (82,165)	<0.001
HDL-cholesterol (mg/dL)	59.7 ± 15.5	50.2 ± 11.8	<0.001	59.7 ± 15.5	50.2 ± 11.8	<0.001
HbA1c (%)	5.6 ± 0.6	6.0 ± 1.0	<0.001	5.6 ± 0.6	6.0 ± 1.0	<0.001
Glucose (mg/dL)	97.9 ± 18.4	111.1 ± 29.2	<0.001	98.7 ± 17.8	112.3 ± 28.6	<0.001
Hs-CRP (mg/dL)	0.2 ± 0.6	0.2 ± 0.3	0.268	0.2 ± 0.6	0.2 ± 0.3	0.306
*Helicobacter pylori* infection, *n* (%)	704 (56.2)	660 (59.7)	0.086	954 (56.2)	896 (59.9)	0.033
Increased arterial stiffness, *n* (%)	402 (32.1)	455 (41.2)	<0.001	533 (32.5)	610 (40.8)	0.001

*Continuous and categorical variables are shown as mean ± SD and numbers (percentages) NAFLD, nonalcoholic fatty liver disease; MAFLD, metabolic dysfunction-associated fatty liver disease; BMI, body mass index; WC, waist circumference; SBP, systolic blood pressure; DBP, diastolic blood pressure; HDL, high-density lipoprotein; HbA1c, glycated hemoglobin; Hs-CRP, high sensitivity C-reactive protein*.

a *Median (interquartile range)*.

### Risk of Arterial Stiffness According to NAFLD/MAFLD and *Hp* Infection

We investigated the risk of increased arterial stiffness according to NAFLD/MAFLD and *Hp* infection. In the univariate analysis, subjects with *Hp* infection but without NAFLD had a significantly higher risk of increased arterial stiffness [odds ratio (OR) 1.33, 95% confidence interval (CI) 1.04–1.69] than subjects without *Hp* infection and NAFLD (used as a control group). Subjects with NAFLD but without *Hp* infection and those with both NAFLD and *Hp* infection also had a significantly higher OR for increased arterial stiffness (OR 1.47, 95% CI 1.12–1.92 and OR 1.95, 95% CI 1.53–2.48, respectively). When adjusting for multiple metabolic factors, including age, BMI, hypertension, diabetes, dyslipidemia, and smoking, the higher risk of increased arterial stiffness in the NAFLD (+) *Hp* (−) and NAFLD (+) *Hp* (+) groups remained (OR 1.61, 95% CI 1.15–2.26 and OR 2.23, 95% CI 1.63–3.06, respectively, Table 2). Regarding MAFLD, subjects with MAFLD but without *Hp* infection and subjects with both MAFLD and *Hp* infection exhibited significantly higher risks of increased arterial stiffness in a dose-dependent manner (OR 1.80, 95% CI 1.36–2.39 and OR 2.13, 95% CI 1.64–2.78, respectively). Meanwhile, in subjects without NAFLD or MAFLD, the risk of arterial stiffness tended to increase in the *Hp* (±) group compared to *Hp* (−), but statistical significance was not observed (OR 1.23, 95% CI 0.92–1.65, *P* = 0.170 and OR 1.16, 95% CI 0.91–1.48, *P* = 0.237, respectively).

**Table 2:** Univariate and multivariate analyses of the risk for arterial stiffness in the total population.

	**Univariate OR**	***P-*value**	**Multivariate OR**	***P-*value**
	**(95% CI)**		**(95% CI)**	
**NAFLD^a^**				
Age	1.17 (1.15–1.18)	<0.001	1.16 (1.15–1.18)	<0.001
Sex	1.14 (0.96–1.35)	0.136		
Hypertension	2.96 (2.49–3.52)	<0.001	2.19 (1.76–2.73)	<0.001
Diabetes	2.78 (2.23–3.47)	<0.001	1.51 (1.14–2.00)	0.004
Dyslipidemia	1.76 (1.49–2.09)	<0.001	1.08 (0.87–1.34)	0.489
Body mass index	0.96 (0.94–0.99)	0.005	0.86 (0.83–0.90)	<0.001
Smoking	1.43 (1.20–1.69)	<0.001	1.93 (1.55–2.40)	<0.001
**NAFLD and** ***HP***				
NAFLD (−) *Hp* (−)	1 (Ref)		1 (Ref)	0.256
NAFLD (−) *Hp* (+)	1.33 (1.04–1.69)	0.021	1.23 (0.92–1.65)	0.170
NAFLD (+) *Hp* (−)	1.47 (1.12–1.92)	0.005	1.61 (1.15–2.26)	0.006
NAFLD (+) *Hp* (+)	1.95 (1.53–2.48)	<0.001	2.23 (1.63–3.06)	<0.001
**MAFLD^b^**				
Age	1.16 (1.15–1.17)	<0.001	1.17 (1.15–1.18)	<0.001
Sex	1.12 (0.95–1.30)	0.181		
Body mass index	0.95 (0.93–0.98)	<0.001	0.89 (0.87–0.92)	<0.001
Smoking	1.44 (1.25–1.66)	0.011	2.07 (1.73–2.47)	<0.001
**MAFLD and** ***HP***				
MAFLD (−) *Hp* (−)	1 (Ref)		1 (Ref)	
MAFLD (−) *Hp* (+)	1.35 (1.10–1.66)	0.004	1.16 (0.91–1.48)	0.237
MAFLD (+) *Hp* (−)	1.49 (1.19–1.87)	0.001	1.80 (1.36–2.39)	<0.001
MAFLD (+) *Hp* (+)	1.85 (1.50–2.27)	<0.001	2.13 (1.64–2.78)	<0.001

*NAFLD, nonalcoholic fatty liver disease; OR, odds ratio; CI, confidence interval; MAFLD, metabolic dysfunction-associated fatty liver disease; Hp, Helicobacter pylori*.

a *Adjusted for age, body mass index, hypertension, diabetes, dyslipidemia, and smoking*.

b *Adjusted for age, body mass index, and smoking*.

When we performed an analysis stratified according to sex, the higher risk of increased arterial stiffness in subjects with both NAFLD and *Hp* infection persisted in both men and women (OR 2.28, 95% CI 1.52–3.42 and OR 2.18, 95% CI 1.29–3.68, respectively, Supplementary Table 1), and similar trends were observed for men and women subjects with MAFLD (OR 1.95, 95% CI 1.43–2.66 and OR 2.66, 95% CI 1.60–4.42, respectively).

### Advanced Fibrosis, *Hp* Infection, and Increased Arterial Stiffness

Next, we performed subgroup analysis in patients with NAFLD/MAFLD for the association between advanced fibrosis, *Hp* infection and increased arterial stiffness. When participants with NAFLD/MAFLD were categorized according to the presence of advanced fibrosis using the FIB-4 index, high FIB-4 index was significantly associated with increased risk of arterial stiffness compared to low FIB-4 index in both patients with NAFLD and MAFLD (NAFLD: OR 2.53, 95% CI, 1.87–3.43, *P* <0.001 and MAFLD: OR 2.95, 95% CI, 2.31–3.76, *P* <0.001). *Hp* infection was independently associated with arterial stiffness in both patients with NAFLD and MAFLD (NAFLD: OR 1.36, 95% CI, 1.04–1.78, *P* = 0.026 and MAFLD: OR 1.31, 95% CI, 1.05–1.64, *P* = 0.016, Table 3).

**Table 3:** Multivariate analyses of the risk for arterial stiffness in subjects with NAFLD/MAFLD.

	**NAFLD^a^**		**MAFLD^b^**	
	**Adjusted OR**	***P-*value**	**Adjusted OR**	***P-*value**
	**(95% CI)**		**(95% CI)**	
Hypertension	2.90 (2.19–3.84)	<0.001		
Diabetes	1.89 (1.39–2.57)	<0.001		
Dyslipidemia	1.19 (0.90–1.58)	0.229		
Body mass index	0.85 (0.82–0.89)	<0.001	0.88 (0.84–0.91)	<0.001
Smoking	1.48 (1.13–1.92)	0.004	1.38 (1.11–1.72)	0.004
High FIB-4 vs. Low FIB-4	2.53 (1.87–3.43)	<0.001	2.95 (2.31–3.76)	<0.001
*Helicobacter pylori*	1.36 (1.04–1.78)	0.026	1.31 (1.05–1.64)	0.016

*NAFLD, nonalcoholic fatty liver disease; MAFLD, metabolic dysfunction-associated fatty liver disease; OR, odds ratio; CI, confidence interval; FIB-4, fibrosis 4 index*.

a *Adjusted for body mass index, hypertension, diabetes, dyslipidemia, and smoking*.

b *Adjusted for body mass index, and smoking*.

## Discussion

To the best of our knowledge, our study is the first to show an interactive effect of NAFLD/MAFLD and *Hp* infection on arterial stiffness. In the present study, a significantly increased risk of arterial stiffness was observed in subjects with NAFLD/MAFLD and *Hp* infection compared with subjects without these conditions. *Hp* infection additively increased the risk of arterial stiffness in subjects with NAFLD or MAFLD.

Arterial stiffness is one of the major indicators of systemic atherosclerosis and is closely related to cardiovascular risk (27). Since increased arterial stiffness is associated with adverse cardiovascular outcomes, even in the general population (28), measurements of arterial stiffness may be helpful to identify high-risk groups for cardiovascular diseases. As a novel indicator of arterial stiffness, CAVI represents the stiffness of the entire arterial segment and is independent of blood pressure, making it highly reproducible and easy to measure (29). Thus, CAVI has been used as a screening tool to evaluate the subclinical atherosclerotic risk in asymptomatic individuals (30). In the present study, we used CAVI as a tool to measure arterial stiffness and revealed that both the presence of NAFLD/MAFLD and *Hp* infection were independently associated with increased arterial stiffness.

Previous studies have investigated the association between NAFLD and increased arterial stiffness. Arterial stiffness indicated by CAVI was associated with the ultrasonography-diagnosed presence and severity of NAFLD (3), and arterial stiffness measured using the augmentation index was associated with more severe NAFLD histology in the biopsy-proven NAFLD cohort (31, 32). NAFLD defined using controlled attenuation parameters also showed a significant association with increased arterial stiffness (24, 33). Consistent with previous results, the presence of NAFLD/MAFLD and advanced fibrosis were independently associated with arterial stiffness in our study.

Several possible mechanisms supporting the association between NAFLD and arterial stiffness are plausible. NAFLD has been recognized as a hepatic manifestation of metabolic syndrome and is closed associated with hyperglycemia, dyslipidemia, and insulin resistance (34), all of which are associated with subclinical inflammation, vascular endothelial cells damage, prothrombotic status, and hemodynamic changes that may increase the risk of atherosclerosis (35). Increased oxidative stress (36), chronic subclinical inflammation (37), reduced levels of adiponectin (38) and altered production of coagulant factors can be involved in the pathogenesis of atherosclerosis in patients with NAFLD.

On the other hand, several studies have suggested that *Hp* infection increases cardiovascular disease, including coronary artery disease and peripheral arterial stiffness (39–41). Choi et al. found that *Hp* seropositivity was significantly associated with increased arterial stiffness (12). Yoshikawa et al. reported that *Hp* infection accelerated the effects of impaired glucose metabolism and increased arterial stiffness (42). Chronic *Hp* infection has been reported to trigger an inflammatory reaction and release inflammatory cytokines, which lead to endothelial dysfunction (43). According to Yu et al. the combination of *Hp* infection and NAFLD increases carotid artery plaque formation, a surrogate marker of atherosclerosis (OR = 1.93). The risk of atherosclerosis was significantly increased in the fatty liver (±) *Hp* (±) group, but not in the fatty liver (±) *Hp* (−) group in the previous study (44). Consistently, *Hp* infection additively increased the risk of arterial stiffness in subjects with NAFLD/MAFLD, and the risk was higher than that of previous study with OR = 2.23 and 2.13 in our study. Moreover, the risk of atherosclerosis showed a dose-dependent relationship in *Hp* (−) NAFLD/MAFLD and *Hp* (±) NAFLD/MAFLD [OR (95% CI), 1.61 (1.15–2.26) and 1.80 (1.36–2.39) in Hp (−) NAFLD/MAFLD vs. 2.23 (1.63–3.06) and 2.13 (1.64–2.78) in Hp (±) NAFLD/MAFLD, respectively]. Collectively, arterial stiffness, measured using CAVI, is a novel approach in the present study, and this association was probably attributed to the synergistic effect of *Hp* infection and NAFLD/MAFLD on atherosclerosis.

Because NAFLD is a sexually dimorphic disease with respect to epidemiological and clinical features (45), we performed an analysis stratified according to sex. The increased risk of arterial stiffness in subjects with both NAFLD/MAFLD and *Hp* infection persisted in both men and women, suggesting the additive effect of *Hp* infection and NAFLD/MAFLD on arterial stiffness in both sexes.

Liver fibrosis is a crucial prognostic factor for cardiovascular outcomes in NAFLD (46). When we evaluated the association between FIB-4 index and increased arterial stiffness in subjects with NAFLD or MAFLD, high FIB-4 index was associated with increased arterial stiffness compared to low FIB-4 index in both NAFLD and MAFLD, suggesting the role of advanced fibrosis in the subclinical atherosclerosis. In line with our results, advanced fibrosis was associated with carotid atherosclerosis in patient with NAFLD (47, 48).

Interestingly, BMI showed an inverse correlation with arterial stiffness in this study. This phenomenon has also been reported in previous studies, and part of this complex association can be explained by the obesity paradox (49, 50). That is, it is explained that some of the patients with elevated BMI benefit from the preservation of arterial stiffness by increased metabolic reserves, attenuated response to renin–angiotensin–aldosterone system, greater muscular strength, potentially protective cytokines and neuroendocrine factors (51, 52). However, there are studies showing that obesity is associated with high CAVI levels and insulin resistance, an independent predictor of vascular stiffness, so additional studies are needed (53, 54).

Our study has several limitations. First, the cross-sectional nature of the study design limits the ability to assess cause and effect. Thus, we were unable to infer causal relationships from this study. Second, the NAFLD diagnosis was exclusively based on ultrasonography, but was not confirmed by liver biopsy, which is the standard diagnostic modality for confirming NAFLD. Ultrasonography has a high specificity but underestimates hepatic steatosis when the fat content is <20% and is unable to quantify fibrosis (55). However, liver biopsy is not typically performed in asymptomatic individuals, and radiographic techniques such as ultrasonography or magnetic resonance imaging are used to diagnose NAFLD in clinical practice. Third, we could not exclude patients with chronic liver disease due to causes other than viral and alcoholic hepatitis. In addition, subjects who take steatogenic drugs could not excluded from this study. However, since this study is based on health check-up examination data targeting asymptomatic adults, the prevalence of this area is thought to be low. Fourth, although the serological test does not discriminate current and past *Hp* infections (56), the *Hp* infection status was assessed only with serology and not other assessment methods, such as the urease breath test or a rapid urease test, in the present study. Due to its cost-effectiveness and invasiveness, serology tests are a common method used in health screening centers that conduct routine blood sampling. We thoroughly investigated the history of *Hp* eradication therapy to supplement the shortcomings of serological tests and overcome this limitation. Fifth, although significant alcohol consumption is considered to be >30 g for men and 20 g for women per day according to the Korean Association for the Study of the Liver Clinical Practice Guideline for NAFLD (57), sex-specific criteria could not be applied to the amount of alcohol consumed in this study. Last, our study population of those who underwent health evaluations upon their own initiative may not represent the majority of the general Korean population, which may contribute to selection bias.

## Conclusions

We demonstrated the synergistic effect of *Hp* infection and NAFLD/MAFLD on the risk of arterial stiffness among asymptomatic Koreans. *Hp* infection additively increases the risk of arterial stiffness in subjects with NAFLD or MAFLD. Therefore, evaluating *Hp* infection status in patients with NAFLD/MAFLD may be helpful in cardiovascular risk assessment. Further studies are needed to determine whether eradication of *Hp* and adequate management of NAFLD/MAFLD helps to improve arterial stiffness and prevent cardiovascular disease.

## Data Availability Statement

The original contributions presented in the study are included in the article/Supplementary Material, further inquiries can be directed to the corresponding author/s.

## Ethics Statement

The studies involving human participants were reviewed and approved by Institutional Review Board of Seoul National University Hospital. Written informed consent for participation was not required for this study in accordance with the national legislation and the institutional requirements.

## Author Contributions

GC: conceptualization. GC, JC, HP, YH, JL, HL, SC, and SL: data curation. JC: formal analysis. SC, JY, and SL: supervision. JC and GC: writing—original draft preparation. All authors have read and agreed to the published version of the manuscript.

## Conflict of Interest

The authors declare that the research was conducted in the absence of any commercial or financial relationships that could be construed as a potential conflict of interest.

## Publisher's Note

All claims expressed in this article are solely those of the authors and do not necessarily represent those of their affiliated organizations, or those of the publisher, the editors and the reviewers. Any product that may be evaluated in this article, or claim that may be made by its manufacturer, is not guaranteed or endorsed by the publisher.
