# Supplementary material for: Non-alcoholic/Metabolic-Associated Fatty Liver Disease and Helicobacter pylori Additively Increase the Risk of Arterial Stiffness
Source: Front Med (Lausanne). 2022 Feb 25;9:844954. doi: 10.3389/fmed.2022.844954 (PMC8914072; doi:10.3389/fmed.2022.844954)
Supplement: Supplementary file 1 [file Table_1.docx]

**Supplementary Table 1.** Multivariate analyses of the risk for arterial stiffness according to sex

|  | Men | |  | Women |  |
| --- | --- | --- | --- | --- | --- |
| NAFLD^a^ | OR (95% CI) | |  | OR (95% CI) |  |
| Age | 1.15 (1.13-1.18) | <0.001 | | 1.18 (1.15-1.21) | <0.001 |
| Hypertension | 1.99 (1.51-2.63) | <0.001 | | 2.56 (1.78-3.68) | <0.001 |
| Diabetes | 1.64 (1.18-2.27) | 0.003 | | 1.31 (0.77-2.23) | 0.325 |
| Dyslipidemia | 1.01 (0.77-1.33) | 0.930 | | 1.20 (0.84-1.71) | 0.315 |
| Body mass index | 0.87 (0.82-0.92) | <0.001 | | 0.86 (0.83-0.90) | <0.001 |
| Smoking | 2.15 (1.64-2.83) | <0.001 | | 0.89 (0.37-2.16) | 0.796 |
| NAFLD and *Hp* |  |  | |  |  |
| NAFLD (-) *Hp* (-) | 1 (Ref) |  | | 1 (Ref) |  |
| NAFLD (-) *Hp* (+) | 1.30 (0.86-1.95) | 0.214 | | 1.18 (0.77-1.82) | 0.450 |
| NAFLD (+) *Hp* (-) | 1.50 (0.97-2.33) | 0.066 | | 2.01 (1.15-3.53) | 0.015 |
| NAFLD (+) *Hp* (+) | 2.28 (1.52-3.42) | <0.001 | | 2.18 (1.29-3.68) | 0.004 |
| MAFLD^b^ |  | |  |  |  |
| Age | 1.16 (1.14-1.18) | | <0.001 | 1.19 (1.16-1.22) | <0.001 |
| Body mass index | 0.89 (0.85-0.92) | | <0.001 | 0.89 (0.84-0.95) | <0.001 |
| Smoking | 2.12 (1.70-2.64) | | <0.001 | 1.45 (0.74-2.84) | 0.275 |
| MAFLD and *Hp* |  | |  |  |  |
| MAFLD (-) *Hp* (-) | 1 (Ref) | |  | 1 (Ref) |  |
| MAFLD (-) *Hp* (+) | 1.15 (0.84-1.57) | | 0.381 | 1.17 (0.79-1.74) | 0.439 |
| MAFLD (+) *Hp* (-) | 1.60 (1.15-2.24) | | 0.006 | 2.40 (1.41-4.11) | 0.001 |
| MAFLD (+) *Hp* (+) | 1.95 (1.43-2.66) | | <0.001 | 2.66 (1.60-4.42) | <0.001 |

NAFLD, nonalcoholic fatty liver disease; OR, odds ratio; CI, confidence interval; MAFLD, metabolic dysfunction-associated fatty liver disease; *Hp*, *helicobacter pylori*

^a^adjusted for age, hypertension, diabetes, dyslipidemia, body mass index, and smoking

^b^adjusted for age, body mass index, and smoking
